# Transient and late-life rapamycin for healthspan extension

**DOI:** 10.18632/aging.102947

**Published:** 2020-03-12

**Authors:** Ellen K. Quarles, Peter S. Rabinovitch

**Affiliations:** 1 University of Michigan, Molecular, Cellular, and Developmental Biology Department, Ann Arbor, MI 48109, USA; 2 University of Washington, Pathology Department, Seattle, WA 98195, USA

**Keywords:** rapamycin, aging, healthspan, persistence, intermittent

Rapamycin is arguably the best-studied pharmaceutical intervention for reliable lifespan and healthspan extension in a wide array of model organisms. These consistent results are encouraging for those eager to develop interventions for prolonging human lifespan or healthspan. Until recently publications of rapamycin treatment in animal models focused on near lifelong treatment, a scenario that is unrealistic to apply to improving the human condition. However, this is beginning to change.

A few groups have endeavored to address this by administering rapamycin to mammalian model organisms beginning at mid- to late-life. Results so far have been encouraging - even when delivered late in life, rapamycin can improve both health- and lifespan in mice [1].

To bring the field even closer to a limited duration regimen that continues to benefit the organism in late life, some groups have published that both intermittent or transient rapamycin treatment can improve lifespan or organ function. Our own work has demonstrated that 8 weeks of rapamycin delivered late in life in mice can confer an improvement in diastolic heart function. This effect persisted for a further 8 weeks post-treatment, even after the metabolic changes due to acute treatment reverted back to pre-treatment levels [2]. Transient (3-month) rapamycin treatment is also sufficient to alter the gut microbiome, alter cancer risk, and significantly improve lifespan when administered to middle-aged mice [3]. That rapamycin could be useful for larger mammals was given credence through a study of dog cardiac outcomes: 10 weeks of rapamycin administered to middle-aged companion dogs was sufficient to improve measures of both systolic and diastolic cardiac function [4]. Human trials of late-life treatment with rapamycin have to date been restricted to the study of immune response and reduction of infection – this work has generated very promising results [5]. These and other studies support the idea that a relatively brief regimen of rapamycin given in late life can confer an improvement to health outcomes, and that these benefits may even persist after treatment ceases.

A critical goal of any pharmaceutical treatment is to minimize off-target effects. Rapamycin’s use in the clinic has been extensive, and side-effects have been reported, though they generally resolve when the drug is removed. Efforts to reduce these off-target responses in humans have ranged from co-treatment with another drug, reducing the dose of rapamycin, and changing the dosing schedule to a more intermittent or transient one [6]. Altering the delivery of rapamycin from continuous to intermittent may help in animal models as well; Apelo and colleagues found that the positive effects of an intermittent rapamycin treatment can be separated from its side effects [7]. At 2mg/kg per day, every five days, beginning at 20 months of age in mice, rapamycin could increase medial and maximal lifespan without detrimental effects on glucose homeostasis. This was also in the absence of metabolic effects seen in models using higher/longer-term doses of rapamycin. Intermittent treatment may, therefore, help to balance the minimization of off-target effects with the desired continual boost to health-span.

At this point, there is a great need to test this drug in similar conditions as it would be translated to clinical use. Lifelong administration has been an extremely useful tool to grow our understanding of mechanisms of aging. But more and more, research indicates that not only do we *not* need to give anti-aging interventions life-long, but this may even be detrimental to the organism, especially early in life -- an example of antagonistic pleiotropy [8]. For the goal of translating geroscience to the betterment of human healthspan, the focus must be on later-life interventions and limiting the time that we actively administer the drug.

## References

[1] Zhang Y, et al. J Gerontol A Biol Sci Med Sci. 2014; 69:119–30. 10.1093/gerona/glt056 23682161 PMC4038246

[2] Quarles E, et al. Aging Cell. 2020; 2:e13086. 31823466 10.1111/acel.13086 PMC6996961

[3] Bitto A, et al. eLife. 2016; 5:e16351. 10.7554/eLife.16351 27549339 PMC4996648

[4] Urfer SR, et al. Geroscience. 2017; 39:117–27. 10.1007/s11357-017-9972-z 28374166 PMC5411365

[5] Mannick JB, et al. Sci Transl Med. 2018; 10:eaaq1564. 10.1126/scitranslmed.aaq1564 29997249

[6] Kaplan B, et al. Transplant Rev (Orlando). 2014; 28:126–33. 10.1016/j.trre.2014.03.002 24685370

[7] Arriola Apelo SI, et al. J Gerontol A Biol Sci Med Sci. 2016; 71:876–81. 10.1093/gerona/glw064 27091134 PMC4906329

[8] Schmeisser K, Parker JA. Front Cell Dev Biol. 2019; 7:192. 10.3389/fcell.2019.00192 31572724 PMC6749033

